# Structure and permeability of the egg capsule of the placental Australian sharpnose shark, *Rhizoprionodon taylori*

**DOI:** 10.1007/s00360-021-01427-0

**Published:** 2022-02-04

**Authors:** Alice L. Buddle, James U. Van Dyke, Michael B. Thompson, Colin A. Simpfendorfer, Christopher R. Murphy, Margot L. Day, Camilla M. Whittington

**Affiliations:** 1 School of Life and Environmental Sciences, The University of Sydney, Heydon-Laurence Building (A08), Sydney, NSW 2006 Australia; 2 Department of Pharmacy and Biomedical Science, School of Molecular Sciences, La Trobe University, Wodonga, VIC Australia; 3 College of Science of Engineering, James Cook University, Townsville, QLD Australia; 4 School of Medical Sciences (Anatomy and Histology), The University of Sydney, Sydney, NSW Australia; 5 Discipline of Physiology, School of Medical Sciences, The University of Sydney, Sydney, NSW Australia

**Keywords:** Chondrichthyan, Pregnancy, Electron microscopy, Ussing chamber, Viviparity

## Abstract

Shark placentae are derived from modifications to the fetal yolk sac and the maternal uterine mucosa. In almost all placental sharks, embryonic development occurs in an egg capsule that remains intact for the entire pregnancy, separating the fetal tissues from the maternal tissues at the placental interface. Here, we investigate the structure and permeability of the egg capsules that surround developing embryos of the placental Australian sharpnose shark (*Rhizoprionodon taylori*) during late pregnancy. The egg capsule is an acellular fibrous structure that is 0.42 ± 0.04 μm thick at the placental interface between the yolk sac and uterine tissues, and 0.67 ± 0.08 μm thick in the paraplacental regions. This is the thinnest egg capsule of any placental shark measured so far, which may increase the diffusion rate of respiratory gases, fetal wastes, water and nutrients between maternal and fetal tissues. Molecules smaller than or equal to ~ 1000 Da can diffuse through the egg capsule, but larger proteins (~ 3000–26,000 Da) cannot. Similar permeability characteristics between the egg capsule of *R. taylori* and other placental sharks suggest that molecular size is an important determinant of the molecules that can be exchanged between the mother and her embryos during pregnancy.

**Supplementary Information:**

The online version contains supplementary material available at 10.1007/s00360-021-01427-0.

## Introduction

Shark reproduction is diverse: some species lay fertilised eggs (oviparous), but most species give birth to fully-developed young (viviparous; Buddle et al. 2019; Hamlett et al. 2005b). The majority of viviparous sharks develop without a placenta and are primarily reliant on yolk for embryonic nourishment during pregnancy (Hamlett et al. 2005b; Buddle et al. 2019). Placentae may form to provide developing embryos with nutrients in addition to the egg yolk in species from five shark families (Carcharhinidae, Sphyrnidae, Hemigaleidae, Leptochariidae and Triakidae) within the order Carcharhiniformes (ground sharks; Hamlett et al. 2005a, b; Buddle et al. 2019).

Regardless of reproductive mode, all shark eggs are fertilised in the oviduct and pass to the uterus through the oviducal gland, where egg coats are added to the fertilised egg (Hamlett et al. 2005b). The oviducal gland is divided into four morphologically and functionally distinct zones: the club, papillary, baffle and terminal zones (Hamlett et al. 1998, 2005b). The club and papillary zones secrete an egg jelly around the fertilised egg, and the baffle zone of most sharks secretes a collagenous capsule that encloses the egg and jelly (exceptions include *Centroscyllium fabricii;* Yano 1995 and *Etmopterus princeps*; Cotton et al. 2015, which are not enclosed by an egg capsule at any stage of pregnancy). The terminal zone stores sperm in some sharks (e.g. *Iago omanensis;* Hamlett 2002; *Mustelus antarcticus;* Storrie et al. 2009 and *Rhizoprionodon taylori;* Simpfendorfer 1992)*.*

In oviparous sharks, the egg capsule is relatively thick, providing mechanical support for the egg, and protects the developing embryos from predators and pathogens in the surrounding seawater (Powter and Gladstone 2008; Awruch 2015). Complex tendrils, ribs and ridges are specific to oviparous egg capsules, and function to secure the eggs to corals, rocks, and crevices on the seafloor (Knight et al. 1996; Heiden et al. 2005; Buddle et al. 2019). In contrast, the egg capsules of viviparous sharks are less complex and thinner than oviparous egg capsules (Lombardi and Files 1993; Heiden et al. 2005).

Some non-placental viviparous sharks (e.g. species of squaliform, squantiniform, orectolobiform and all species of lamniform sharks) hatch out of their egg capsule during development, which likely facilitates fetomaternal exchange between the embryos and the maternal uterus (Ellis and Otway 2011; Conrath and Musick 2012; Awruch 2015). Conversely, almost all placental sharks remain in their egg capsule until birth (Hamlett et al. 2005a; Buddle et al. 2019). The egg capsule is incorporated into the placental interface and separates the fetal yolk sac from the maternal uterine tissue, except in *Prionace glauca* and *Scoliodon laticaudus* (Buddle et al. 2019, 2021; Hamlett et al. 2005a). Hence, in most placental sharks, transport between the maternal and fetal tissues during pregnancy must occur across the egg capsule (Hamlett et al. 2005a; Buddle et al. 2021). The adaptive significance of egg capsule retention in placental sharks is unclear.

The ability of high and low molecular weight molecules to move through the egg capsule has been investigated in two placental sharks: the dusky smooth-hound shark (*Mustelus canis;* Lombardi and Files 1993) and the bonnethead shark (*Sphyrna tiburo;* Heiden et al. 2005)*.* In both species, the egg capsule is an acellular, collagenous structure that has no visible pores and is ~ 4.7 μm thick in *M. canis* and ~ 1 μm thick in *S. tiburo* (Heiden et al. 2005). Molecules ranging in size from ~ 180 to ~ 1000 Da readily diffuse across the egg capsules of both species, whereas larger proteins (~ 3496–200,000 Da) cannot (Lombardi and Files 1993; Heiden et al. 2005)*.* Therefore, the egg capsule of placental sharks may be permeable only to relatively small molecules (Lombardi and Files 1993; Heiden et al. 2005).

**Fig. 1:**
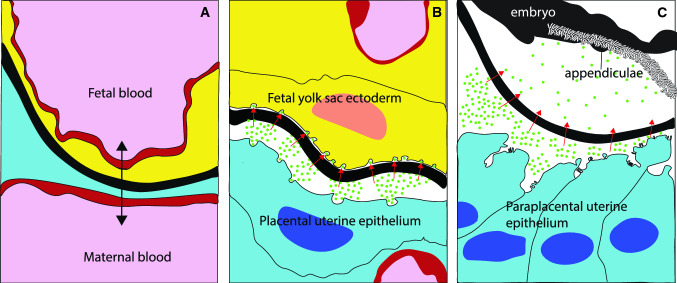
Diagram of proposed mechanisms for transport across the placental and nonplacental egg capsule during late pregnancy in *Rhizoprionodon taylori*. Uterine and placental morphology of *R. taylor*i females is based on Buddle et al. (2021) and the diagram in **B** is adapted from Hamlett (1993). **A** The egg capsule at the placental interface likely allows the diffusion (double arrow) of respiratory gases, water and fetal wastes directly between closely associated fetal and maternal blood streams. **B** In other placental regions, uterine secretions (green circles) are released into the luminal space between the uterine epithelium and the egg capsule. The apposing fetal yolk sac cells may absorb these uterine secretions (red arrows). **C** Uterine secretions released by the paraplacental uterine epithelium may diffuse across the non-placental regions of the egg capsule (red arrows). Subsequently, embryos may ingest or absorb these secretions on the appendiculae of their umbilical cord. **A**–**C** show fetal and maternal blood (pink); endothelial cells of fetal and maternal blood vessels (red); fetal yolk sac ectoderm (yellow); placental and paraplacental uterine epithelium (blue); uterine secretions (green); uterine luminal fluid (white); egg capsule (black) (colour figure online)

Here, we provide the first description of the structure and permeability of the egg capsule of the Australian sharpnose shark, *R. taylori. Rhizoprionodon taylori* suspends embryonic development from the blastodisc stage for 7 months of the 11.5-month pregnancy (Simpfendorfer 1992, 1993). Most embryonic growth occurs rapidly after the placenta forms at 9 months into pregnancy (Simpfendorfer 1992, 1993). Any maternally derived molecule that is required for embryonic development must pass across the egg capsule to reach the developing embryo (Buddle et al. 2021). During late pregnancy, the egg capsule separates the paraplacental uterine epithelium from the luminal fluid and intervenes between the placental uterine epithelium and the fetal yolk sac (Fig. 1; Buddle et al. 2021). Paraplacental uterine secretions may contain nutrients that diffuse across the egg capsule into the uterine fluid for ingestion by the embryos or absorption by the fetal outgrowths on the umbilical cord termed ‘appendiculae’ (Fig. 1; Buddle et al. 2021). At the placental interface, respiratory gases, water, fetal wastes, and potentially small nutrients likely diffuse between fetal and maternal blood streams across the fetal tissues, egg capsule and uterine tissues (Fig. 1; Buddle et al. 2021). The permeability of the egg capsule to molecules is influenced by capsular thickness and structure (Lombardi and Files 1993; Heiden et al. 2005). Since fetomaternal exchange occurs across the entire egg capsule in both placental and paraplacental regions (Buddle et al. 2021), we expected the structure and thickness to be similar across all capsular regions. To allow for fetomaternal exchange of respiratory gases, water, wastes and nutrients during pregnancy, we expected that the egg capsule of late pregnant *R. taylori* is permeable to at least some molecules. Since the size of the molecule is an important determinant of capsular permeability in other placental sharks, we predicted that the egg capsule of *R. taylori* is permeable to relatively small dye molecules and peptides (< ~ 1000 Da), but not larger proteins (> 3000 Da).

## Methods

### Sample collection

Five late-pregnant female *R. taylori* were collected in December 2019 using monofilament gillnets in Cleveland Bay, north Queensland, Australia. Sharks were immediately euthanised after capture by severing the cervical spinal cord with a sharp knife. The two uteri were dissected out of each female. Each uterus contained two to four embryos, with five to seven total embryos per female. The uterine wall was opened to expose the uterine lumen and the egg capsules surrounding the individual embryos. Egg capsules were cut near the cranial end of each embryo and peeled away from the uterine wall. The egg capsule at the placental interface was tightly interdigitated between maternal and fetal tissues and could not be separated intact from these tissues. Therefore, we were unable to isolate the part of the egg capsule that is incorporated into the placenta, and these sections were preserved intact between maternal and fetal tissues (see below). All other non-placental parts of the egg capsule were removed and rinsed several times in elasmobranch Ringer’s solution (Babkin et al. 1933). One egg capsule from each female was processed for scanning and transmission electron microscopy. Four egg capsules from each female were stored at 4 °C in elasmobranch physiological saline solution (Hara et al. 2018) prior to use in the permeability experiments described below.

All research activities were conducted under permits from the Great Barrier Reef Marine Park Authority (G15/37987.1) and Queensland Department of Primary Industries and Fisheries (187,250 and 200,906). The protocol was approved by the James Cook University Animal Ethics Committee (permit no. A2310) and the University of Sydney Animal Ethics Committee (permit no. 2019/1583).

### Scanning electron microscopy

Three portions (each 1 cm^2^) of each egg capsule were dissected and placed in 2.5% glutaraldehyde in 0.1 M phosphate buffer for 1 h. Samples were then rinsed in 0.1 M phosphate buffer and fixed in 1% osmium tetroxide (OsO_4_). After further rinsing in 0.1 M phosphate buffer, samples were gradually dehydrated from 70 to 100% ethanol and then dried with a Leica EM CPD300 Critical Point Dryer (Leica) using carbon dioxide as the drying agent. Dried egg capsules were mounted onto aluminium stubs and coated with gold (15 nm). Samples were viewed and images captured on a JEOL NeoScope JCM-600 Tabletop scanning electron microscope and a Zeiss Sigma HD VP STEM (Zeiss).

### Transmission electron microscopy

Three pieces (~ 1 cm^2^) of each of the egg capsules were dissected and fixed in 2.5% glutaraldehyde in 0.1 M phosphate buffer as per the scanning electron microscopy samples. Egg capsule pieces were then rinsed in 0.1 M phosphate buffer and fixed in 1% osmium tetroxide (OsO_4_) with 1% potassium ferrocyanide in 0.1 M phosphate buffer at room temperature for 1 h. Samples were rinsed in 0.1 M phosphate buffer and then dehydrated in a series of ethanol. Ethanol was gradually replaced by Spurr’s resin (Agar Scientific, Essex, UK) in 25% increments. Egg capsule pieces were then transferred to individual BEEM capsules and polymerised at 60 °C overnight. Ultrathin sections (70 nm) were cut using a Ultracut S (Leica, Wetzlar, Germany) microtome with glass knives and placed on 200 mesh copper grids. Three to five grids were made per resin block. Grids were post stained with 2% uranyl acetate for 10 min, rinsed three times in deionised warm water and then stained with Reynold’s lead citrate stain surrounded by sodium hydroxide pellets for 10 min. Grids were rinsed three times with deionised warm water and then air dried. Sections were viewed and imaged with a FEI Tecnai T12 Transmission Electron Microscope (TEM) at 80 kV.

### Egg capsule thickness measurements

Four transmission electron micrographs (magnification 5200 ×) of different regions of one egg capsule from each of four females were selected to determine the thickness of the egg capsule in paraplacental regions. To measure the thickness of the egg capsule at the placental interface, four transmission electron micrographs (magnification 5200 ×) of the placental interface from four different females were used. Ten measurements were taken per image, and thickness was measured using ImageJ software.

The mean and standard error of the mean (SEM) were calculated for each female using the 40 measurements taken for each female (4 micrographs per females, 10 measurements per micrograph). Mean egg capsule thicknesses in paraplacental regions (n = 4) were compared to the thicknesses of the egg capsules at the placental interfaces (n = 4) using an unpaired t-test. A *p* value less than 0.05 was deemed significant.

Two scanning electron micrographs of different regions of one egg capsule from each of two females were oriented to allow an opportunistic estimate of egg capsule thickness to confirm the transmission electron micrograph measurements.

### Permeability of the egg capsule

To investigate the permeability characteristics of the egg capsule in *R. taylori*, we used a Ussing chamber system (Fig. S1; Lombardi and Files 1993; Heiden et al. 2005)*.* For each permeability experiment, five portions (2–3 cm^3^) were dissected from five egg capsules from each of five females. A single layer of egg capsule was stretched onto a 1.26 cm^2^ slider and mounted into an EasyMount Ussing chamber system (Fig. S1; Physiologic Instruments, San Diego, CA, United States). The egg capsule was surrounded with 2 mL of elasmobranch saline solution (Hara et al. 2018) on each side of the chamber (Fig. S1).

To determine the permeability of the egg capsule to bromocresol green, 50 µL of elasmobranch saline solution containing 1 mM concentration of bromocresol green (698 Da) was added to side A of chamber (Fig. S1). At two timed intervals (30 min and 1 h), 200 µL of solution was taken from side B of the chamber (Fig. S1). Solutions were analysed on a CLARIOstar Plus microplate reader (BMG Labtech) in absorbance mode with a reading wavelength set to 423 nm. The same experimental design was used to independently determine the permeability of the egg capsule to 1 mM concentration of rose bengal (1,018 Da) in elasmobranch saline solution. The reading wavelength for analysing the presence of rose bengal was set to 549 nm.

Five μL of the ‘Ultra-low Range Molecular Weight Marker’ (M.W. 1060–26,600 Da; Sigma- Aldrich, St Louis, Missouri) containing 0.4–0.6 mg of triosephosphate isomerase from rabbit muscle (26,600 Da), myoglobin from horse heart (17,000 Da), α-Lactalbumin from bovine milk (14,200 Da), aprotinin from bovine lung (6500 Da), Insulin Chain B, oxidized from bovine pancreas (3496 Da) and bradykinin (1060 Da) was added to 10 μL of elasmobranch saline solution and vortexed. This protein solution was then added to the 2 mL of elasmobranch saline solution on side A of the chamber (Fig. S1). The chamber was covered and left at room temperature for 1 h. All solutions from both sides (A and B) of the chamber were collected and immediately stored at − 20 °C prior to gel electrophoresis. The proteins in the samples were concentrated and dried using a SpeedVac (Genevac miVac Duo, Fisher, CITY, Spain). Precipitated proteins were then dissolved in sample buffer (100 mM Tris–HCl, pH 6.8, 1% SDS, 4% 2-mercaptoethanol, 0.02% Brilliant Blue G, and 24% glycerol) and heated at 65 °C for 2 min. Protein samples were separated at 150 V for 1.5 h on mini-PROTEAN 16.5% polyacrylamide Tris-Tricine precast gel (Bio-Rad Laboratories, Inc. Hercules, CA) electrophoresis. Gels were then rinsed in deionised water and fixed in 5% glutaraldehyde in deionised water for 1 h. Gels were rinsed again in deionised water and stained with Coomassie blue (EZblue gel staining reagent, Sigma-Aldrich, USA) overnight. After staining, gels were rinsed with deionised water and imaged using a Chemidoc MP imaging system (Bio Rad Laboratories, Hercules, CA). To check that the Brilliant Blue G dye in the sample buffer (854 Da) was completely removed from the gel during the dying process, we separated and then fixed and dyed gels with just the sample buffer under the same conditions as the protein samples.

## Results

During late pregnancy, individual *R. taylori* embryos, and their associated umbilical cords and placentae, are separated from each other in the uterus by folds in the uterine mucosa that form compartments (Fig. S2). The egg capsule is filled with luminal fluid and lies opposed to the paraplacental regions of these uterine compartments (Fig. S2). The placenta consists of a portion of the uterine wall, the egg capsule and the fetal yolk sac (Fig. S2). The portion of the egg capsule that is incorporated into the placenta is highly folded and cannot be separated from the uterine and fetal tissues.

**Fig. 2:**
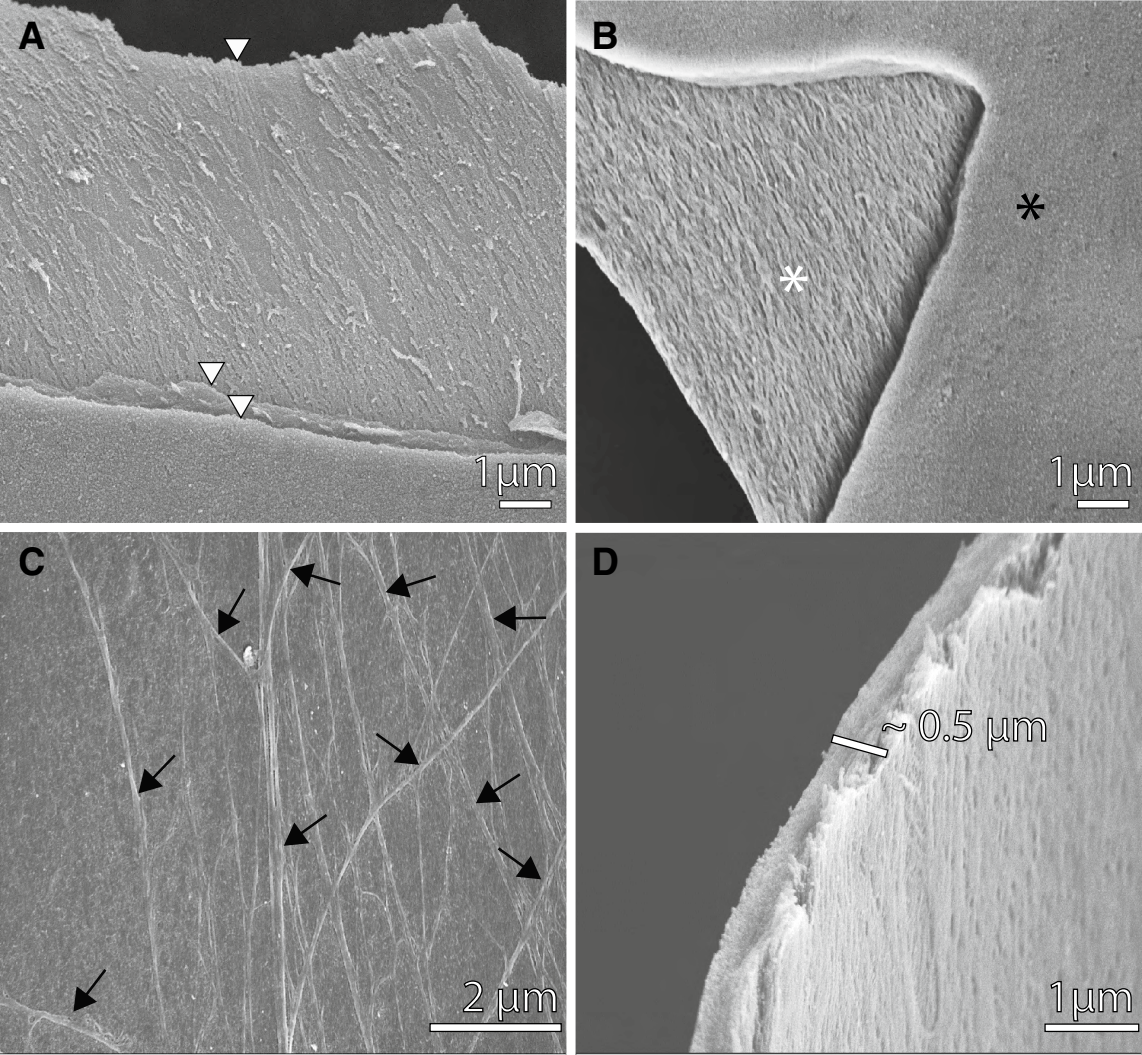
Scanning electron micrographs of the egg capsule in non-placental regions of late pregnant *Rhizoprionodon taylori*. **A** The egg capsule consists of three fibrous layers (white arrowheads). **B** The surface layer (black asterisk) is smooth compared to the underlying layers (white asterisk). **C** Fibres running in different directions (black arrows) are evident on the surface of some regions of the egg capsule. **D** The thickness of the egg capsule layers is approximately 0.5 μm

The egg capsule that is opposed to the paraplacental uterus of *R. taylori* is composed of three fibrous layers (Fig. 2A). Each layer is composed of fibres that are evident in the layers that underlie the mostly smooth surface layer (Fig. 2B). None of the layers has visible pores (Fig. 2). Branching fibres occur on the surface of some regions of the egg capsule (Fig. 2C). The egg capsule is 0.5 ± 0.04 μm thick (Fig. 2D).

**Fig. 3:**
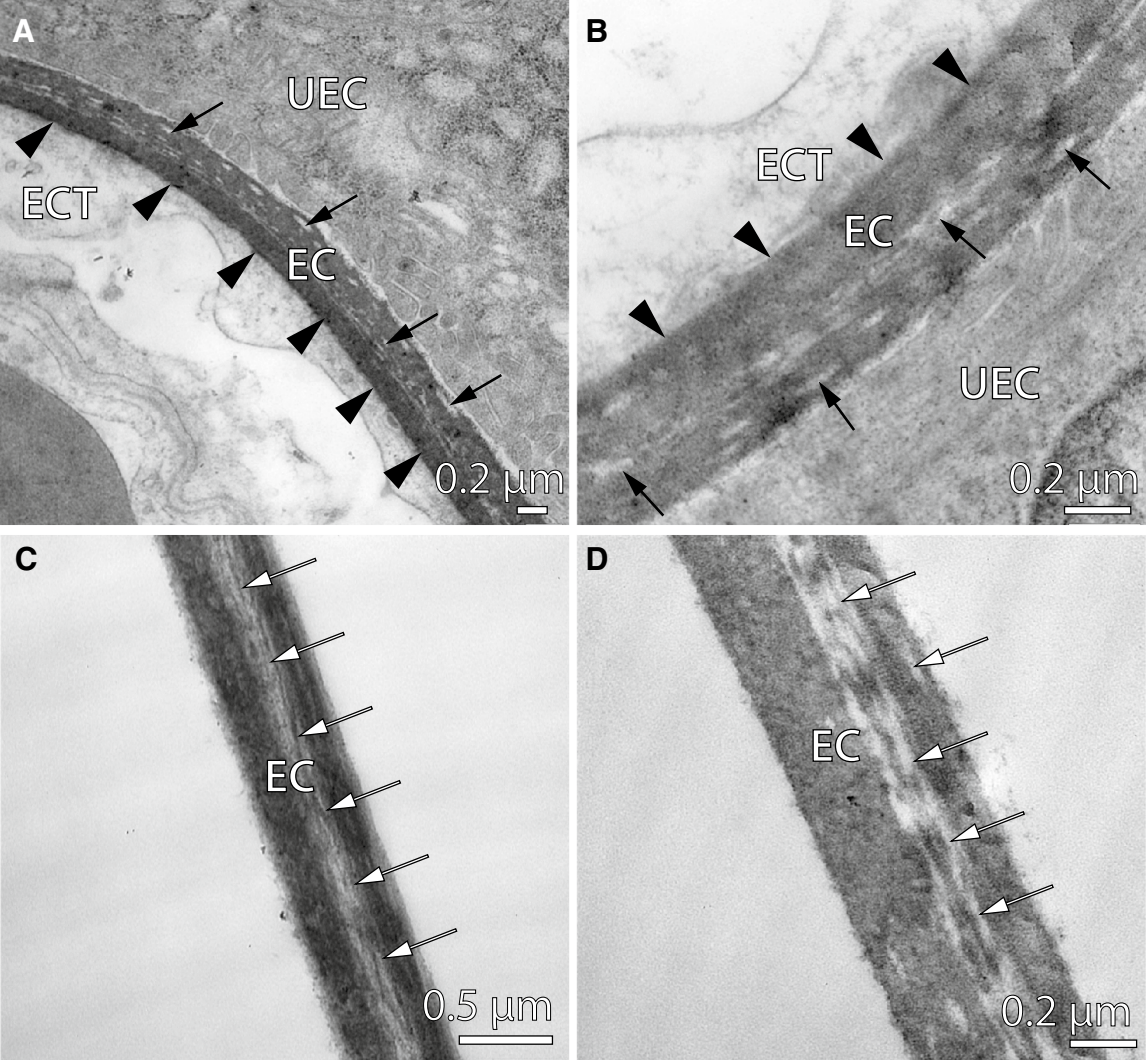
Transmission electron micrographs of the egg capsule in non-placental and placental regions of late pregnant *Rhizoprionodon taylori*. **A**, **B** The egg capsule (EC) adheres to the fetal yolk sac ectodermal cells (ECT) at the placental interface (black arrowheads). Gaps (arrows) in the egg capsule layers are evident on the side of the egg capsule that faces the uterine epithelial cells (UEC). **C**, **D** The non-placental egg capsule is unattached the fetal yolk sac tissue and gaps (arrows) in the egg capsule layers also occur

The egg capsule at the placental interface of late pregnant *R. taylori* adheres to the yolk sac ectodermal cells (Fig. 3A, B). The layer of egg capsule that is attached to these fetal cells is solid, whereas striations occur in the egg capsule layers that face the uterine epithelium (Fig. 3A, B). In all other paraplacental regions around the embryo, the egg capsule is unattached to the fetal yolk sac tissue (Fig. 3C, D). Spaces in the egg capsule layers are evident in the paraplacental regions of the egg capsule, which is similar to the egg capsule in the placental regions (Fig. 3C, D).

The thickness of the egg capsule between the uterine epithelium and the fetal yolk sac ectoderm at the placental interface is 0.42 ± 0.04 μm. In the paraplacental regions, the egg capsule is ~ 0.2 μm thicker (0.67 ± 0.08 μm) than in the placental regions. This paraplacental egg capsule thickness is similar to our measurement of the egg capsule in the paraplacental region using scanning electron micrographs (Fig. 2D). There was no significant difference between the mean thickness of the egg capsule in placental and paraplacental regions (*t*_6_ = 2.236, *p* = 0.067).

**Fig. 4:**
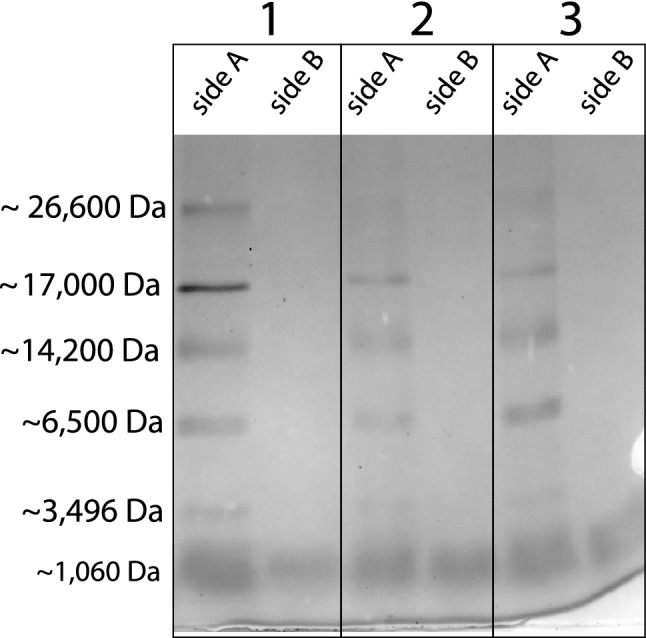
SDS-PAGE gel stained with coomassie blue showing the protein permeability of the egg capsule in the non-placental regions of late pregnant *Rhizoprionodon taylori*. Sections 1, 2 and 3 represent separate Ussing chamber systems and are representative subsets of all trials. We added the protein mixture to Side A, which shows each protein represented by a single band at the correct molecular weight (1060–26,600 Da). The band at 1060 Da in the side B lanes indicates that bradykinin crossed the egg capsule after 1 h. The lack of bands in the side B lanes for proteins (3496–26,600 Da) demonstrate that these proteins did not cross the egg capsule after 1 h

After 30 min and 1 h, respectively, both bromocresol green (698 Da) and rose bengal (1000 Da; Table 1) dyes had passed through the egg capsule surrounding *R*. *taylori* embryos*.* All proteins [triosephosphate isomerase from rabbit muscle (26,600 Da); myoglobin from horse heart (17,000 Da); α-Lactalbumin from bovine milk (14,200 Da); aprotinin from bovine lung (6500 Da) and Insulin Chain B, oxidized from bovine pancreas (3496 Da) and bradykinin (1060 Da)] were detected as a single band in the solution samples collected from Side A of the Ussing chamber (Fig. 4). The presence of a single band at 1060 Da indicates that only bradykinin (1060 Da) crossed through the egg capsule to side B of the Ussing chamber after 1 h (Fig. 4). The absence of all other bands in the solution samples from side B of the Ussing chamber indicates that the five other proteins ranging from 3496 to 26,000 Da did not diffuse through the egg capsule after 1 h (Fig. 4). The lack of bands in the gels with only the sample buffer added to each well indicated that Brilliant G dye was successfully removed from the gels during the gel dying process (data not shown).

**Table 1:** Permeability of dyes and proteins across the egg capsule of *Rhizoprionodon taylori* (this study), *Sphyrna tiburo* (Heiden et al. 2005) and *Mustelus canis* (Lombardi and Files 1993)

Molecule	Species		
	*R. taylori*	*S. tiburo*	*M. canis*
Urea (60 Da)			Yes
Glucose (180 Da)		Yes	Yes
Tyrosine (181 Da)		Yes	
Cresol red (382 Da)		Yes	
Thymol blue (466 Da)		Yes	
Bromothymol blue (624 Da)		Yes	
Bromocresol green (698 Da)	Yes	Yes	
Methylthymol blue (756 Da)		Yes	
Fast green fcf (809 Da)			Yes
Rose bengal (1018 Da)	Yes		Yes^a^
Bradykinin (1060 Da)	Yes^a^		
Vitamin B12 (1355 Da)		Yes^a^	
Insulin (3496 Da)	No		No
Aprotinin (6500 Da)	No	No	
α-Lactalbumin (14,200 Da)	No		
Lysozyme (14,300 Da)		No	No
Myoglobin (17,000 Da)	No		
Trypsin inhibitor (21,500 Da)		No	
Triosephosphate isomerase (26,000 Da)	No		
Carbonic anhydrase (31,000 Da)		No	
Lactate dehydrogenase (36,500 Da)		No	
Glutamic dehydrogenase (55,400 Da)		No	
Bovine serum albumin (66,300 Da)		No	No
Phosphorylase b (97,400 Da)		No	
Beta galactosidase (116,300 Da)		No	
Myosin (200,000 Da)		No	

Blank spaces indicate that molecule has not been investigated in that species. Molecular weights for the dye molecules are sodium-free

^a^The largest molecule to pass through the egg capsule in each species

## Discussion

The acellular egg capsule that is incorporated into the placental interface of late pregnant *R. taylori* is similar in structure to the egg capsule that lies opposed to the paraplacental uterine epithelium. The placental regions of the egg capsule are not significantly thinner (0.42 ± 0.04 μm) than the paraplacental regions (0.67 ± 0.08 μm), but our relatively small sample size may have limited our ability to detect a significant difference between them. Slight differences in capsular thickness may be due to the adherence of the egg capsule to the fetal yolk sac ectodermal cells, which only occurs at the placental interface. The fetal yolk sac cells that oppose the placental uterine epithelial cells are extremely thin in some regions (Buddle et al. 2021). The thin fetal and uterine cell layers reduce the diffusion distance for respiratory gas exchange across the egg capsule between the closely associated fetal and maternal blood streams at the placental interface (Buddle et al. 2021). In the paraplacental regions, the egg capsule is not in direct contact with the fetal yolk sac tissue but lies opposed to the paraplacental columnar uterine epithelium, which is morphologically specialised for secretion. The paraplacental egg capsule may be thin enough to allow for paraplacental uterine secretions to pass through. Therefore, the relatively consistent thickness of the egg capsule surrounding late pregnant *R. taylori* embryos likely facilitates fetomaternal exchange between fetal and uterine tissues in both placental and paraplacental regions.

In paraplacental regions, the egg capsule of *R. taylori* is permeable to low molecular-weight dyes (bromocresol green 698 Da and rose bengal 1018 Da) and the peptide bradykinin (1060 Da), but larger proteins (3496–26,000 Da) do not diffuse across the egg capsule. This result supports the hypothesis that the egg capsule in placental sharks is permeable only to low molecular weight molecules (Lombardi and Files 1993; Heiden et al. 2005). Unfortunately, separating the egg capsule from the fetal yolk sac and the uterine tissues at the placental interface of late pregnant *R. taylori* was impossible without tearing the capsule, because the tissues are so strongly interdigitated, and the yolk sac adheres to the egg capsule. Therefore, the permeability of the egg capsule in placental regions remains unmeasured. Given that the structure of the egg capsule appears similar under TEM regardless of region, we assume that all regions of the egg capsule are permeable to similarly-sized small molecules. If the diffusion rate of molecules across the egg capsule is inversely proportional to the thickness of the capsule (following Fick’s First Law of Diffusion), then diffusion should be faster across the thinner egg capsule in the placental regions than the thicker egg capsule in the paraplacental regions.

The egg capsule layers surrounding the embryos of *R. taylori* are considerably thinner (0.4–0.6 μm) than the egg capsules of other placental sharks (*S. tiburo* 4.7 μm; Lombardi and Files 1993 and *M. canis* 1 μm; Heiden et al. 2005). Therefore, the barrier to exchange between fetal and uterine tissues is less significant in *R. taylori* than in *M. canis* and *S. tiburo.* This difference may arise due to the different requirements of *R. taylori* embryos compared to other placental sharks. Unlike the gradual embryonic development that occurs through a 4.5–5-month pregnancy in *S. tiburo* and an 11-month pregnancy in *M. canis,* in *R. taylori*, the 7-month embryonic diapause means that all embryonic growth occurs rapidly in the final 3–4 months of pregnancy (Simpfendorfer 1992, 1993)*.* The demand for efficient exchange of respiratory gases, water, fetal wastes, and nutrients between fetal and maternal tissues increases as embryos grow during pregnancy (Ferner and Mess 2011; Sato et al. 2016). Therefore, *R. taylori* embryos may have a thinner egg capsule than *M. canis* and *S. tiburo* embryos to facilitate rapid growth in the final few months of pregnancy. Alternatively, differences between species may be due to different stages of embryonic development examined in each species, or the different methods used to assess capsular thickness; we used transmission electron micrographs to measure the thickness of the egg capsule in both placental and paraplacental regions of *R. taylori,* whereas only the paraplacental regions of the egg capsule were measured by light micrographs in *S. tiburo* (Heiden et al. 2005) and one scanning electron micrograph in *M. canis* (Lombardi and Files 1993). Future research should use transmission electron microscopy to measure the thickness of egg capsules in both placental and paraplacental regions of a wide range of placental sharks. If *R. taylori* has a considerably thinner egg capsule than other placental sharks, then egg capsule thickness may be determined by species-specific embryonic requirements during pregnancy.

The egg capsule of *R. taylori* consists of at least three fibrous layers, which are visible on the surface of the egg capsule. Multiple fibrous layers of egg capsule surround the embryos of other placental sharks (*M. canis;* Lombardi and Files 1993; *S. tiburo;* Heiden et al. 2005)*,* but the number of egg capsule layers differs between species; *S. tiburo* also has three layers (Heiden et al. 2005), whereas the egg capsule of *M. canis* has four layers (Lombardi and Files 1993). A higher number of egg capsule layers increases the overall thickness of the egg capsule; *M. canis* has a considerably thicker egg capsule (4.7 μm; Lombardi and Files 1993) than both *S. tiburo* (1 μm; Heiden et al. 2005) and *R. taylori* (0.4–0.6 μm). The umbilical cord of *R. taylori* and *S. tiburo* is covered in ‘appendiculae’ outgrowths, which are morphologically specialised for absorption (Buddle et al. 2021; Schlernitzauer and Gilbert 1966)*,* while *M. canis* has a smooth umbilical cord that lacks these outgrowths (Cateni et al. 2003). Appendiculae may absorb histotrophic nutritive secretions from the paraplacental uterine epithelium (Hamlett 1989). Placental sharks that lack umbilical cord appendiculae such as *M. canis* probably rely on nutrient transfer across the placenta rather than by absorbing paraplacental uterine secretions from the uterine fluid via the umbilicus (Hamlett 1989). Therefore, the reduction in the egg capsule layers of *R. taylori* and *S. tiburo* may reduce the barrier between the paraplacental uterine epithelium and the uterine fluid to facilitate the transfer of nutritive paraplacental secretions for absorption by the appendiculae (Heiden et al. 2005). Future work should determine the structure and number of egg capsule layers in other placental sharks that both possess and lack appendiculae on their umbilical cord. If all placental sharks with umbilical cord appendiculae have fewer and thinner egg capsule layers than species that lack these structures, then egg capsule layers may be associated with absorption of paraplacental uterine secretions by appendiculae.

Given that the egg capsule remains intact around developing embryos at all stages of pregnancy, any maternal nutrients supplied to *R. taylori* embryos in addition to the egg yolk must pass through the capsule to reach the fetal tissues (Buddle et al. 2021). If molecular weight determines which materials can cross the egg capsule, low molecular weight urea (60 Da) and nutrients such as inorganic ions, water, glucose (180 Da), and most amino acids (75–204 Da) and free fatty acids (~ 278–338 Da) should readily diffuse across the capsule from maternal to fetal tissues. However, the ability of molecules to diffuse through the egg capsule of *R. taylori* may be influenced by more factors than just molecular weight. For example, the hydrophilic/lipophilic properties and the conjugated system of the molecule influence whether a molecule can pass through the egg coats that surround mammalian embryos during early development (Turner and Horobin 1997; Denker 2000). Most metabolites and biologically active compounds can pass through mammalian egg coats (Turner and Horobin 1997; Denker 2000). While we did not test the ability of specific metabolites and nutrients to diffuse across the egg capsule, we speculate that most molecules required for embryonic development would pass through, given that embryonic growth is so rapid during late pregnancy in *R. taylori*. Any nutrient that is not able to diffuse across the egg capsule could be provided by the embryo’s egg yolk. Quantifying the major classes of nutrients in the egg yolk of *R. taylori* will help elucidate the contribution of the egg yolk, paraplacenta and placenta to embryonic nourishment during pregnancy.

Larger proteins ranging in molecular weight from 3496 to 26,600 Da did not cross the egg capsule of *R. taylori* after 1 h, which suggests that the molecular cut-off weight is between ~ 1060 Da (bradykinin) and 3496 Da (Insulin Chain B, oxidized from bovine pancreas). Proteins that range in size from 6500 to 200,000 Da are not able to diffuse across the egg capsule of *S. tiburo* after 1 h or 24 h, which suggests that the permeability of the egg capsule to proteins is not time dependent (Heiden et al. 2005). The ability of molecules smaller than ~ 1355 Da to diffuse through the egg capsule of *R. taylori, M. canis* and *S. tiburo* suggests that the molecular cut-off size of the egg capsule of all placental sharks is around 1355 Da (Table 1). Testing this hypothesis requires data on the permeability of the egg capsule from a wider range of placental shark species.

The permeability of the egg capsule only to low molecular weight materials suggests that maternal nutrient transfer to developing *R. taylori* embryos involves uterine histotrophic secretion of relatively small molecules such as sugars (monosaccharides or disaccharides), amino acids and fatty acids for transfer across the egg capsule. Hemotrophic transfer of nutrients between fetal and maternal blood streams may occur across the egg capsule in placental regions where fetal and maternal blood vessels are less than 2 μm apart (Buddle et al. 2021). Since the egg capsule of *R. taylori* is impermeable to proteins equal to or larger than 1060 Da, the diffusion of nutrients directly between fetal and maternal blood streams is likely to be limited to small organic molecules and inorganic ions. Support for this hypothesis could be provided by the localisation of transporter proteins involved in small organic and inorganic nutrient transfer to placental regions where fetal and maternal blood vessels are extremely close (Buddle et al. 2021).

Steroid hormones such as progesterone, estrogens and testosterone are relatively small lipid-soluble molecules (~ 300 Da; Hunter 2012) and therefore, should be able to move across the egg capsule of *R. taylori* between uterine and fetal cell layers due to their small sizes. The decline in progesterone levels (0.02–0.85 ng ml^−1^) in the maternal plasma after the embryonic diapause period, and during the placental embryonic growth period in *R. taylori*, suggests that progesterone is involved in maintaining diapause by preventing embryonic development (Waltrick et al. 2014). Testosterone levels (0.53–7.09 ng ml^−1^) are elevated in the maternal plasma during late pregnancy in *R. taylori* and may be involved in triggering the transition from lecithotrophic yolk only nourishment to uterine histotrophic fetal nourishment during mid to late pregnancy (Waltrick et al. 2014). The human placenta produces and maintains the steroid hormones (progesterone and estrogen) circulating in the maternal blood during pregnancy (Napso et al. 2018). Golgi and rough endoplasmic reticulum in the fetal yolk sac cells and the uterine cells at the placental interface of *R. taylori* and other placental sharks (Hamlett et al. 2005a; Buddle et al. 2021), suggest that the fetal and/or maternal portions of the placenta may be involved in secreting hormones during pregnancy. If the fetal tissues secrete testosterone during pregnancy, then developing *R. taylori* embryos may be able to manipulate the maternal uterine tissues to produce nutritive secretions during pregnancy (Haig 1996). The relatively large size of polypeptide and protein hormones (e.g. prolactin, growth hormone and placental lactogen ~ 22,000 Da; Norman et al. 2015) that are released by the fetal tissues of mammals and act on the receptors of their mother (Haig 2008), may preclude the involvement of these types of hormones in feto-maternal signalling across the egg capsule of *R. taylori.* Support for this hypothesis would be provided by an absence of genes involved in fetal prolactin and growth hormone production, and their maternal receptors, in the placental tissues of *R. taylori.*

The inability of larger molecules to diffuse across the egg capsule of late pregnant *R. taylori* may serve to protect developing embryos during pregnancy, as egg coats do in other species. For example, the acellular zona pellucida egg coat of rats is permeable to much larger proteins (e.g., peroxidase, 40,000 Da and ferritin, 500,000 Da; Hastings et al. 1972) than the egg capsule of *R. taylori* and other placental sharks (Table 1; ~ 1000 Da; Heiden et al. 2005; Lombardi and Files 1993). Despite this permeability to such large proteins, the zona pellucida may still prevent potential pathogens such as viruses and bacteria from passing to the embryo during early pregnancy (Eaglesome et al. 1980; Van Soom et al. 2010). Given that viruses and bacteria are orders of magnitude larger than ~ 1000 Da*,* the egg capsule of placental sharks may have a similar protective function through pregnancy.

Furthermore, given that the *R. taylori* egg capsule physically separates genetically-different fetal and maternal tissues through pregnancy, it may also serve to protect fetal tissue from recognition by the maternal immune system (Denker 2000; Menkhorst and Selwood 2008). Like mammals, fetal-maternal immune interactions during pregnancy may be facilitated by natural killer cells and lymphoid aggregates in the uterine wall of the placental shark, *Rhizoprionodon terraenovae* (Haines et al. 2006)*.* The cytokine interlukin-1 system may also be involved in mediating fetal-maternal immune reactions during pregnancy in *M. canis* (Haines et al. 2006)*.* Cytokines are larger (~ 6000–70,000 Da; Stenken and Poschenrieder 2015) than the largest molecule (~ 1000 Da) that diffuses across the egg capsule of all placental sharks investigated so far (Table 1; Heiden et al. 2005; Lombardi and Files 1993). Therefore, the mechanisms involved in feto-maternal communication by cytokines are unclear in placental sharks and require further investigation. Retention of the egg capsule at the placental interface of sharks may facilitate adhesion between fetal and maternal tissues during placental formation in *M. canis* because glycans are associated with the egg capsule, fetal yolk sac cells and opposing placental uterine epithelial cells (Jones and Hamlett 2004)*.* Therefore, the egg capsule of *R. taylori* and most other placental sharks may serve as an immunological barrier between the fetal tissue and the maternal tissues through the entire pregnancy. Three placental sharks (*Scoliodon laticaudus, Prionace glauca* and *Iago omanensis;* Hamlett et al. 2005a) have lost their egg capsules, providing an excellent opportunity to test the hypothesis of an immunoprotective function for the egg capsule. If the egg capsule is involved in preventing the rejection of fetal tissues during placental formation in most placental sharks with an egg capsule, then sharks that lack an egg capsule at the placental interface (*S. laticaudus, P. glauca* and *I. omanensis*) must have different mechanisms that allow for placental formation during pregnancy. These mechanisms may include those that are involved in regulating immune responses during mammalian pregnancy such as a reduction in maternal leukocytes (e.g., macrophages, uterine natural killer cells and regulatory T-cells) as pregnancy progresses and the secretion of cytokines (Ander et al. 2019).

In conclusion, the structure and permeability of the acellular egg capsule surrounding the embryos of late pregnant *R. taylori* is similar to other placental sharks (*M. canis;* Lombardi and Files 1993 and *S. tiburo;* Haines et al. 2006), which suggests that the egg capsules of placental sharks allow for selective transport of molecules based on size. Future research should test whether the physiochemical properties of molecules influence the ability of molecules to cross the egg capsule. The egg capsule surrounding late pregnant *R. taylori* embryos is the thinnest of any placental shark egg capsule investigated so far, which suggests that egg capsule thickness may be determined by species-specific embryonic requirements during pregnancy. Thinner egg capsules should allow for more efficient diffusion of respiratory gases, fetal wastes, water and nutrients (e.g., inorganic ions, glucose, amino acids and small fatty acids) across the capsular surface during pregnancy. Determining the expression and localisation of proteins involved in transporting specific molecules across the egg capsule of the shark placenta is an essential next step in understanding feto-maternal exchange during shark pregnancy.

## Supplementary Information

Below is the link to the electronic supplementary material.

Fig. S1. Diagram of the Ussing chamber system used to determine the permeability of the egg capsule to dyes and proteins in late pregnant *Rhizoprionodon taylori.* A portion of the egg capsule was placed between two chambers. 2 mL of elasmobranch saline solution was added to both sides (A and B) of the chamber. Depending on the experiment, dyes or a protein mixture were added to side A of the chamber. Solution from side B of the chamber was collected and analysed for the presence of dyes or proteins. (PDF 40 kb)

Fig. S2. Diagram of *Rhizoprionodon taylori *embryos developing in their egg capsules during late pregnancy adapted from Buddle et al., 2019. Embryos are attached to their placenta by an umbilical cord that is covered in outgrowths termed ‘appendiculae’. Uterine compartments form around each individual embryo by folds in the uterine mucosa. The egg capsule is filled with uterine fluid and lies opposed to the paraplacental uterine epithelium. Additionally, the egg capsule separates the placental portion of the uterine wall from the fetal yolk sac. (PDF 84 kb)

## Data Availability

Data are published in the main manuscript.
