# Supplementary figures and images for: Structure and permeability of the egg capsule of the placental Australian sharpnose shark, Rhizoprionodon taylori
Source: J Comp Physiol B. 2022 Feb 4;192(2):263–73. doi: 10.1007/s00360-021-01427-0 (PMC8894161; doi:10.1007/s00360-021-01427-0)

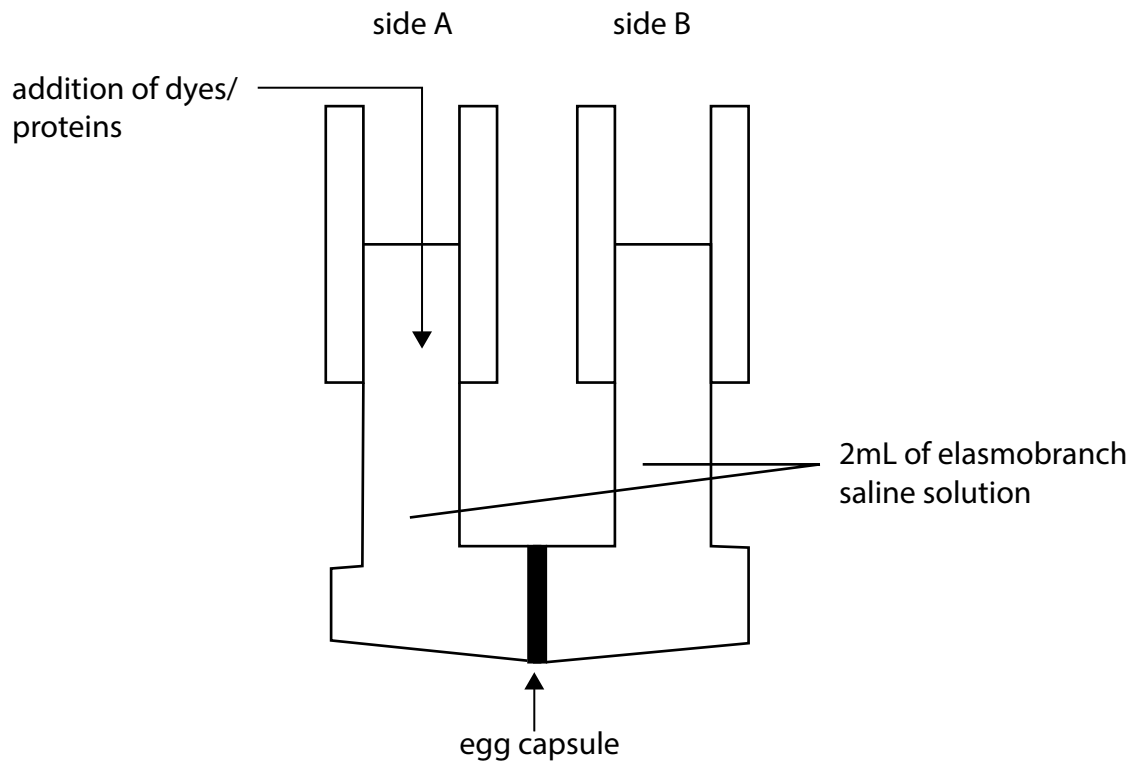

Supplement: Supplementary file 1 — Fig. S1. Diagram of the Ussing chamber system used to determine the permeability of the egg capsule to dyes and proteins in late pregnant Rhizoprionodon taylori. A portion of the egg capsule was placed between two chambers. 2 mL of elasmobranch saline solution was added to both sides (A and B) of the chamber. Depending on the experiment, dyes or a protein mixture were added to side A of the chamber. Solution from side B of the chamber was collected and analysed for the presence of dyes or proteins. (PDF 40 kb) [file 360_2021_1427_MOESM1_ESM.pdf]

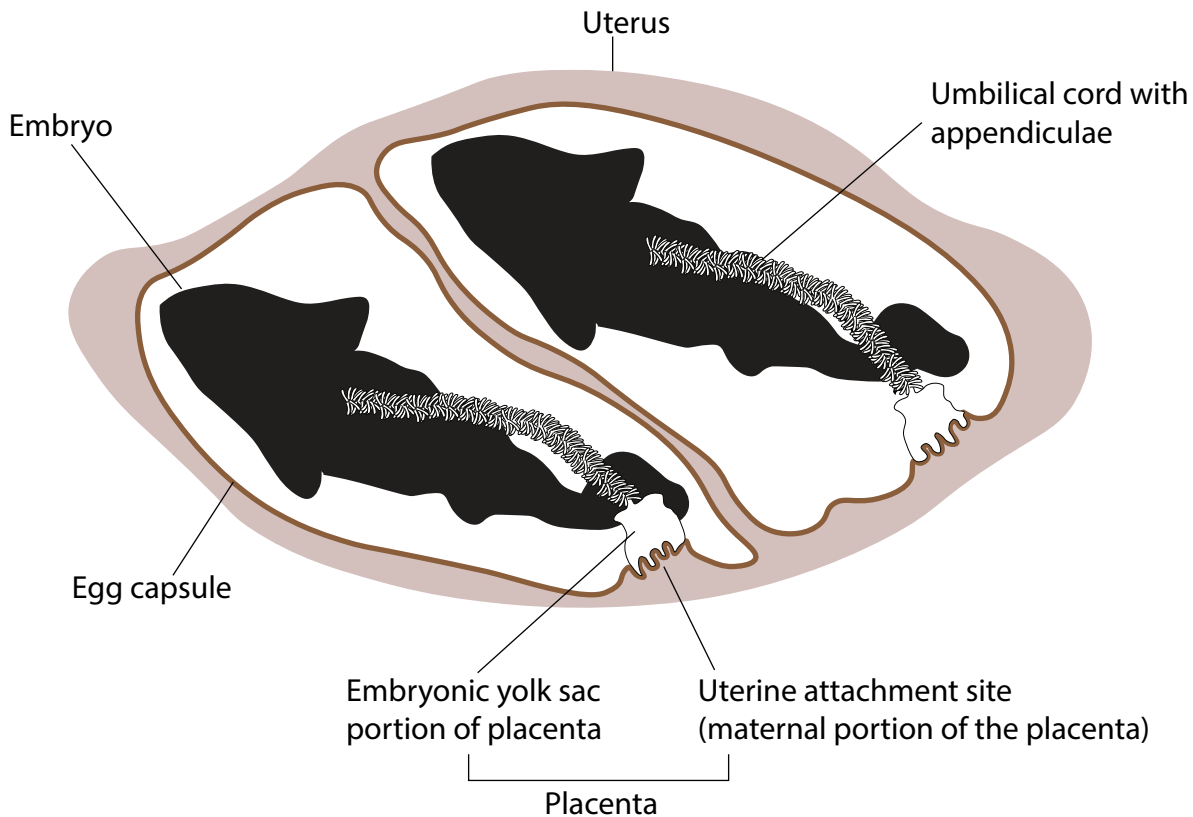

Supplement: Supplementary file 2 — Fig. S2. Diagram of Rhizoprionodon taylori embryos developing in their egg capsules during late pregnancy adapted from Buddle et al., 2019. Embryos are attached to their placenta by an umbilical cord that is covered in outgrowths termed ‘appendiculae’. Uterine compartments form around each individual embryo by folds in the uterine mucosa. The egg capsule is filled with uterine fluid and lies opposed to the paraplacental uterine epithelium. Additionally, the egg capsule separates the placental portion of the uterine wall from the fetal yolk sac. (PDF 84 kb) [file 360_2021_1427_MOESM2_ESM.pdf]
